# Work in progress on infections, reproductive sexual health, education and non-communicable diseases

**DOI:** 10.4314/ahs.v25i3.1

**Published:** 2025-09

**Authors:** James K Tumwine

*Twenty-five years old and going strong:* Twenty-five years ago, we were privileged to start, in a humble way, the maiden issue of *African Health Sciences* (AHS). AHS has since grown into a gold open access journal, in which the authors and readers are not charged fees. We most sincerely thank the friends and partners of AHS, through the Africa Journals partnership (AJPP), for your sustained support as we struggle to push African publishing to greater heights. Our volunteers in and outside Uganda, the authors, reviewers, readers and editors deserve a pat on the back. AHS has now embarked on a new and more focused path, as we face limited resources and the challenges posed by AI. While we embrace the ethical and professional use of AI, we must never forget the importance of RI (Real intelligence).

Our September menu: In this September issue of AHS we bring you very interesting papers on infectious diseases, sexual and reproductive health, education, non-communicable diseases, and surgery. In the following paragraphs, we give you what we have traditionally been calling Editor's choice.

*Infections predominate:* The first paper reports the “effects of genetic polymorphism in susceptibility to schistosomiasis infection and adverse treatment outcomes”^1^. This is followed by one important case control study on factors influencing the development of tuberculosis in Nigeria^2^. Next come findings of a study from Mali reporting on the seroprevalence of anti-SARS-CoV-2 IgG antibodies among vaccinated healthcare workers^3^. The next paper is on the “effect of modified iliac bone graft combined with antibiotic bone cement on lower limb bone infection.”^4^.

From the Democratic Republic of the Congo there is a paper calling for action to curb a serious monkey pox^5^ epidemic. This joins a long list of those conscious of the international dangers of these viral epidemics and their protentional devastating effects as Covid-19 rudely taught us. A paper on factors associated with respiratory tract infection in patients after surgery under general anesthesia^6^ makes this infectious disease section.

*Sexual reproductive health:* The next section highlights sexual and reproductive health issues. It begins with a report on hematologic markers of Systemic Inflammatory Response (SIR) as reliable biomarkers in detecting pre-eclampsia^7^. The next two papers highlight issues of prenatal care: “predictors of traditional prenatal service uptake among rural Zimbabwean” pregnant women^8^; and factors associated with utilization of antenatal care services among urban refugee adolescents in Uganda^9^. Another case control study establishes the link between polycystic ovary syndrome and systemic immune inflammation and other inflammation markers^10^; hence crowning this section on reproductive health.

*Education and diagnosis:* Papers on the value of MRI in the diagnosis of intracranial micro-hemorrhage^11^ together with one on inter-rater reliability of the Glasgow Coma Scale^12^ among health workers, introduce us to this education section. The education theme continues with papers on “bridging the biostatistics gap” in African health research^13^. Then we have another paper on knowledge of sickle cell disease^14^. One paper on the effect of family empowerment health education on health behavior of young stroke patients^15^ crowns this section.

*NCDs:* We then delve into the non-communicable disease arena with papers on factors associated with clinical breast examination and cervical cancer screening uptake in Ghanaian women^16^. Is there evidence of circulating Elabela-21 and Elabela-11 levels as predictive biomarkers in type 2 diabetes? Well, read the paper by Al-Rubaye and others^17^ and find out.

Elderly patients with chronic obstructive pulmonary disease are not easy to nurse. Researchers from China have developed a model-based nursing intervention^18^ on these patients. Read about the impact of their model on self-management ability and quality of life.

Many institutions are introducing electronic medical records. Nigerian scientists have written an interesting paper on “perception and barriers to electronic medical record use among physicians and nurses” in general hospitals^19^. Continuing with the education and health systems theme, we bring you a paper on the ‘effect of continuous professional development and clinical imaging guidelines on reducing inappropriate computerized tomography utilization^20^ among children and young patients in low resource settings.”

*Others:* We end this treatise with seminal papers from Surgery. There is one on “assessment of pain intensity using summed pain intensity difference (SPID) in orthopedic operative patients^21^. In a meta-analysis, another paper gives very interesting update on the effect of balance training with stroboscopic glasses on postural stability and activity level in patients^22^. We end this September menu with a paper on the “clinical effect of comprehensive nursing on patients with slow transit constipation after laparoscopic surgery^23^.”

We wish you enjoyable and fruitful reading through this diverse menu of *African Health Sciences*.
